# Examination of the genetic factors underlying the cognitive variability associated with neurofibromatosis type 1

**DOI:** 10.1038/s41436-020-0752-2

**Published:** 2020-02-04

**Authors:** Myrthe J. Ottenhoff, André B. Rietman, Sabine E. Mous, Ellen Plasschaert, Daniela Gawehns, Hilde Brems, Rianne Oostenbrink, ENCORE-NF1 Team, Marie-Claire Y. de Wit, Pieter F. A. de Nijs, Jeroen S. Legerstee, Gwendolyn C. Dieleman, Leontine W. ten Hoopen, Rick van Minkelen, Mark Nellist, Elizabeth Schorry, Eric Legius, Henriette A. Moll, Ype Elgersma

**Affiliations:** 1 Department of Neuroscience, Erasmus Medical Center, Rotterdam, The Netherlands; 2 Department of Pediatrics, Erasmus Medical Center–Sophia Children’s Hospital, Rotterdam, The Netherlands; 3 The ENCORE Expertise Center for Neurodevelopmental Disorders, Erasmus Medical Center, Rotterdam, The Netherlands; 4 Department of Child and Adolescent Psychiatry and Psychology, Erasmus Medical Center–Sophia Children’s Hospital, Rotterdam, The Netherlands; 5 Department of Human Genetics, KU Leuven, Leuven, Belgium; 6 Department of Clinical Genetics, Erasmus Medical Center, Rotterdam, The Netherlands; 7 Department of Pediatrics, Cincinnati Children’s Hospital Medical Center, Cincinnati, OH USA

**Keywords:** neurofibromatosis type 1, phenotypic variability, genotype–phenotype association, intelligence quotient, twin study

## Abstract

**Purpose:**

Neurofibromatosis type 1 (NF1) is an autosomal dominant disorder associated with cognitive deficits. The NF1 cognitive phenotype is generally considered to be highly variable, possibly due to the observed T2-weighted hyperintensities, loss of heterozygosity, NF1-specific genetic modifiers, or allelic imbalance.

**Methods:**

We investigated cognitive variability and assessed the contribution of genetic factors by performing a retrospective cohort study and a monozygotic twin case series. We included data of 497 children with genetically confirmed NF1 and an IQ assessment, including 12 monozygotic twin and 17 sibling sets.

**Results:**

Individuals carrying an *NF1* chromosomal microdeletion showed significant lower full-scale IQ (FSIQ) scores than individuals carrying intragenic pathogenic *NF1* variants. For the intragenic subgroup, the variability in cognitive ability and the correlation of IQ between monozygotic NF1 twin pairs or between NF1 siblings is similar to the general population.

**Conclusions:**

The variance and heritability of IQ in individuals with NF1 are similar to that of the general population, and hence mostly driven by genetic background differences. The only factor that significantly attenuates IQ in NF1 individuals is the *NF1* chromosomal microdeletion genotype. Implications for clinical management are that individuals with intragenic *NF1* variants that score <1.5–2 SD below the mean of the NF1 population should be screened for additional causes of cognitive disability.

## INTRODUCTION

Neurofibromatosis type 1 (NF1, OMIM 162200) is a genetic disorder (birth prevalence ~1:2000)^1^ caused by heterozygous loss-of-function variants in *NF1* (OMIM 613113). *NF1* encodes the protein neurofibromin, which is a negative regulator of the RAS signaling pathway. Hallmark NF1 features are café-au-lait spots, inguinal freckling, (sub)cutaneous neurofibromas, plexiform neurofibromas, hamartomas of the iris, optic pathway gliomas, and bone dysplasia.^2^ In addition to these somatic features, NF1 individuals have cognitive deficits, behavioral problems, and motor difficulties.^3,4^ The cognitive deficits significantly impact the daily lives of NF1 individuals, as cognitive deficits are rated the highest disease burden in childhood.^5–7^ Additionally, the prevalence of attention deficit–hyperactivity disorder and autism spectrum disorder is increased tenfold in the NF1 population.^5,8,9^

NF1 is inherited in a Mendelian manner with complete penetrance. Nonetheless, individual NF1 characteristics are highly variable in severity and occurrence. Family studies confirmed this phenotypic variability for somatic characteristics such as café-au-lait spots, optic pathway glioma, and various types of neurofibromas by showing low heritability among first and second degree affected family members^10,11^ and even monozygotic (MZ) twins.^12,13^ These findings suggest that a noninherited mechanism drives the etiology of somatic disease characteristics. Indeed, genetic and molecular studies reveal somatic second-hit variants in the unaffected *NF1* allele (leading to loss of heterozygosity [LOH]) in many NF1-related lesions.^14^

The cognitive phenotype is assumed to be highly variable as well, which has led researchers to propose several mechanisms that could cause this variability. This includes mechanisms that are determined by one’s genetic makeup, such as specific effects of certain pathogenic *NF1* variants (genotype–phenotype effects)^15^ or variants in other genes that either worsen or ameliorate the effect of the pathogenic *NF1* variant (genetic modifiers).^15,16^ Additionally, mechanisms that are noninherited and individual-specific, such as LOH^15^ and the presence of T2-weighted hyperintensities (T2Hs; lesions often found on magnetic resonance images [MRIs] of the brains of NF1 individuals), could contribute to the variation.^17,18^ Lastly, it has been proposed that allelic imbalance could play a role,^19–21^ which could involve inherited or noninherited mechanisms.^22^ A known genotype–phenotype correlation has been reported for individuals who carry large deleletions of *NF1* that also affect neighboring genes (so-called chromosomal microdeletions [CMDs]), which results in reduced cognitive ability.^23,24^ However, since patients with a CMD represent only a small portion of the NF1 patient population, it cannot explain the presumed variability of the cognitive phenotype. The relation between intragenic genotypes and cognitive ability has only been investigated in terms of the presence of learning problems as a dichotomous outcome, with one study suggesting a lower cognitive ability in NF1 individuals with pathogenic splicing variants relative to the general NF1 population^25^ and other studies collectively suggesting a higher cognitive ability for individuals carrying a specific pathogenic small in-frame deletion (p.Met992del).^26–29^

Although several mechanisms have been put forward to explain the cognitive variability in NF1, it is notable that clinical studies specifically addressing the extent of the cognitive variability are lacking. A few family studies did include a dichotomous variable for learning problems and showed significant familial clustering.^10–12,30^ However, the measurement as well as the power of that study are insufficient to reliably estimate variability, and the data could be supportive of either normal cognitive variability or high variability due to a dominant role of genotype–phenotype correlations. Hence, we characterized the variability of cognitive ability in one large cohort of children with NF1 from two independent tertiary referral centers for NF1, and analyzed the effects of different types of pathogenic *NF1* variants. Additionally, we assessed variability of IQ within NF1 MZ twin pairs and NF1 sibling sets to further quantify the contribution of noninherited factors and genetic background to cognitive ability in NF1. We chose full-scale IQ (FSIQ) as the primary outcome, as it is highly standardized to yield a normal distribution with a specific mean (100) and standard deviation (15) in the general population, allowing us to place our findings in the context of the variability of IQ in the general population.

## MATERIALS AND METHODS

For the retrospective cohort study, we collected data from patients at two NF1 tertiary referral centers (the Erasmus Medical Center [EMC] Rotterdam, the Netherlands, and the University Hospital Leuven [UHL] Belgium) who had undergone an IQ assessment. Such an assessment is part of the standard follow-up procedure for all NF1 children visiting the expertise centers at EMC and UHL. Although we encourage all children with NF1 in the Netherlands and Belgium to visit our centers, it is possible that a minority of the participants visited our center because of behavioral or cognitive issues, or because they were interested in participating in one of the clinical trials that were performed in our centers^31,32^ or the ongoing trial (clinicaltrials.gov: NCT02256124). All patients were born between 1990 and 2013, had visited the NF1 outpatient clinic at least once before January 2018, and had completed IQ testing before the age of 18 (total *n* = 615). Patients were excluded if (1) a genetic (*n* = 97) or (2) clinical NF1 diagnosis (according to the National Institutes of Health [NIH] diagnostic criteria for NF1^9^) was missing or incomplete (*n* = 10), (3) individuals had segmental NF1 (*n* = 8), (4) a genetically confirmed Legius syndrome was diagnosed (*n* = 3), or (5) a concurrent genetic diagnosis other than NF1 was present affecting cognition or behavior, in case this was documented in the individual’s medical record (*n* = 0). Data on the most recent assessment of FSIQ (primary outcome), verbal and performance IQ (VIQ, PIQ; secondary outcomes) were collected. IQs were assessed by a licensed psychologist as part of a standardized cognitive and behavioral testing battery using an age-appropriate Wechsler Intelligence Scale.

For the MZ twin case series, we screened the NF1 patient registry of the EMC for MZ twin pairs with a genetically confirmed clinical NF1 diagnosis, and no symptomatic brain pathology. Eight of nine pairs at the EMC gave consent for IQ assessment for the purpose of prospective data collection. In addition, retrospective data from four MZ NF1 twin pairs from UHL and Cincinnati Children’s Hospital Medical Center (CCHMC) were included. Like the EMC and UHL, the CCHMC is also a tertiary referral center for NF1 individuals. All twin pairs were confirmed to be MZ with an identifier test (AmpFlSTR Identifiler PCR Amplification Kit, ThermoFisher Scientific) and their IQs were assessed using an age-appropriate Wechsler scale. The study was initially designed to include 29 NF1 MZ twin pairs in order to have enough power (α = 0.05, β = 0.80) to acknowledge an FSIQ intraclass correlation (*r*_ICC_) of 0.615 as significantly lower than that of MZ twins in the general population (*r*_ICC_ = 0.85).^33^ We chose this decrease in *r*_ICC_ for the power analysis, as it is close to the *r*_ICC_ for dizygotic twins (0.60),^33^ and therefore indicates a meaningful decrease. We went slightly above this level to inflate the sample size. However, after including 12 twin pairs we were prompted to stop further inclusion, as the *r*_ICC_ 95% confidence interval (CI) excluded the hypothesized *r*_ICC_ of 0.615. Data on VIQ and PIQ were missing for some retrospective twin cases; therefore, only FSIQ was assessed for the twin case series. Data collection for the retrospective data collection for the cohort study and twin case series was performed with the approval of the EMC Medical Ethics Review Committee, the Ethics Committee Research UZ/KU Leuven, and the CCHMC Institutional Review Board. Additionally, the EMC Medical Ethics Review Committee approved prospective data collection for the twin case series. Written informed consent was obtained from all participants (and from both parents in case of minor participants) who were recruited for prospective data collection within the twin case series. For retrospective data collection, all centers' ethics committees exempted the study from the requirement of receiving informed consent from patients. Prospective data collection for the twin case series was registered in a trial register (clinicaltrials.gov: NCT02436746).

Molecular *NF1* screening and assignment of pathogenicity was performed in a diagnostic setting. The molecular techniques used and the method for assigning pathogenicity have been described previously.^11^ Pathogenic variants were classified using the following variant type categories: missense, frameshift, splicing, nonsense, miscellaneous intragenic (small [16 amino acids] in-frame insertions and deletions and large in-frame and out-of-frame insertions and deletions) and CMDs, including types I–III and atypical deletions. For intragenic variants, these categories were assigned based on their predicted coding effect using a visual variant analysis software package (Alamut Visual, version 2.11). The Leiden Open Variation Database (LOVD, version 3.0, https://databases.lovd.nl/shared/genes/NF1) was consulted for each variant to check if variant-specific RNA-based or protein-based molecular analyses were published that overruled the in silico predicted coding effect. It should be noted that such additional information is only present for a small subset of variants in LOVD, meaning that it is possible that some variants have been misclassified (e.g., classified as missense variants instead of as splicing variants).

### Statistical analysis

Shapiro–Wilk tests were performed to determine whether IQ scores were normally distributed. Linear mixed models (LMMs) with random center effects that accounted for within-center nesting were used to assess the effect of genotype and of variant location along the gene on IQ score. Main effects of fixed variables in LMMs were tested using likelihood ratio tests. For these fixed effects, we additionally calculated standardized effect sizes (*f*^2^), for which a value of ≥0.02 is considered small, ≥0.15 medium, and ≥0.35 large.^34^ Model assumptions for LMMs were checked by visualization of model residuals (residuals versus fitted values plot for homoscedasticity and Q–Q plots for normality). Significant main effects of categorical variables containing >2 levels were followed up by post hoc testing, which was corrected for multiple comparisons using the false discovery rate (FDR). Variance between genotype groups was compared using Levene’s tests. Intraclass correlations (*r*_ICC_) of IQ scores within MZ twin pairs or sibling sets were calculated from a LMM with FSIQ as the dependent variable, age as fixed effect and intercepts per twin pair or sibling set as random effects. We estimated the *r*_ICC_ 95% CIs using 500 bootstrap samples. Descriptive statistics were obtained with contingency table analyses or Mann–Whitney *U* tests depending on variable type. The significance level was set to α = 0.05 (two-sided). Statistical analyses were performed using R (v3.5.1, R foundation), supplemented with the nlme package for LMM analysis,^35^ emmeans package for LMM post hoc analysis,^36^ and gabtools package for extracting *r*^2^ values from LMMs for calculating effect sizes.^37^

## RESULTS

We included 497 children: 394 from the EMC and 103 from the UHL. The cohorts did not significantly differ in sex (proportion female: 46% vs. 54%; χ^2^[1] = 2.00; *p* = 0.16), age at IQ assessment (median = 9 [interquartile range, IQR] = 6–12] vs. 8 [6–12]; *U* = 20,580; *p* = 0.82), FSIQ (mean = 87.1 [SD = 15.4] vs. 86.6 [14.8]; *t*[491] = −0.31; *p* = 0.76) or variant type (χ^2^[5] = 8.87; *p* = 0.11) and were therefore analyzed together.

**Fig. 1:**
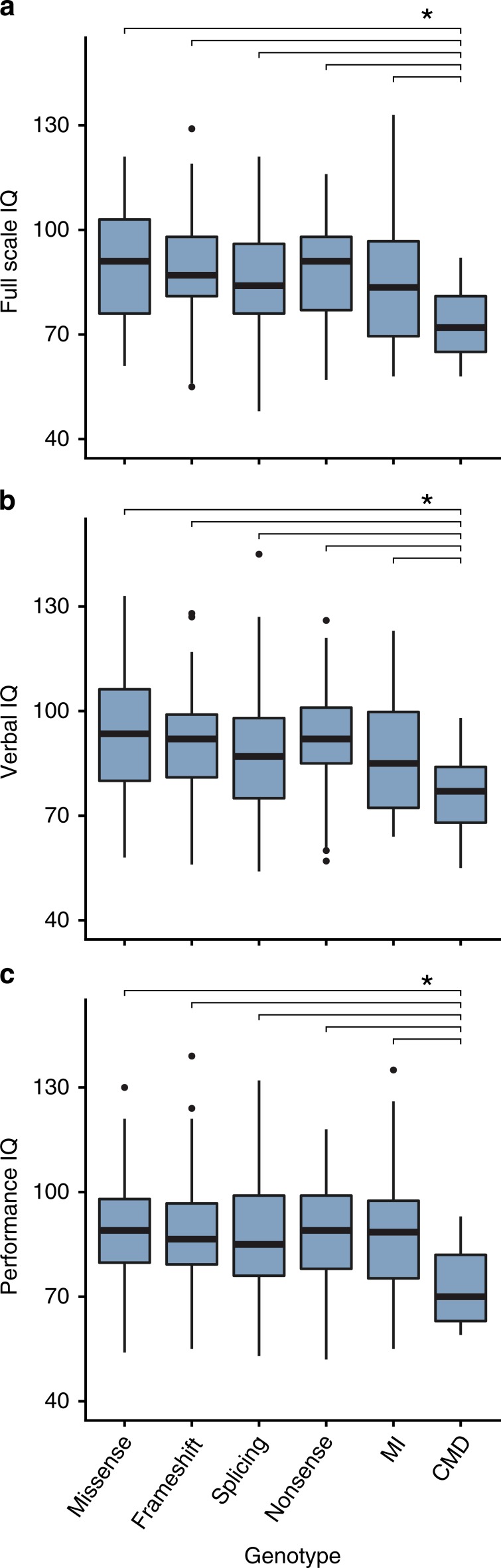
Relationship between neurofibromatosis type 1 (NF1) genotype and IQ. Boxplots show the median and interquartile ranges of full-scale IQ (FSIQ) (**a**), verbal IQ (VIQ) (**b**), and performance IQ (PIQ) (**c**) divided over the different genotype groups. The horizontal bars and asterisk (*) indicate comparisons that remain significant after false discovery rate (FDR)-corrected multiple comparisons. *CMD* chromosomal microdeletion, *MI* miscellaneous intragenic.

To assess if we could confirm a lower cognitive ability for the CMD genotype, we divided the total cohort over six different genotype groups: missense (*n* = 72), frameshift (*n* = 134), splicing (*n* = 97), nonsense (*n* = 129), miscellaneous intragenic (*n* = 38), and CMD (*n* = 25) (Table 1). The main effect of genotype was significant for all three IQ scores: FSIQ (likelihood ratio [*LR*][5] = 27.60; *p* < 0.001; *f*^2^ = 0.054), VIQ (*LR*[5] = 26.82; *p* < 0.001; *f*^2^ = 0.053), and PIQ (*LR*[5] = 23.62; *p* < 0.001; *f*^2^ = 0.046). Subsequent post hoc testing for FSIQ revealed that the group effect was indeed driven by the CMD group, which had significantly lower IQ scores compared with the other groups (Fig. 1 and Table S1).

**Table 1:** IQ scores per genotype.

Genotype	Subjects (*n* = 495)	Mean FSIQ (SD)	Mean VIQ (SD)	Mean PIQ (SD)
Missense	72	89.3 (15.9)	92.9 (18.2)	89.1 (13.4)
Frameshift	134	88.8 (13.7)	91.6 (13.8)	88.1 (15.1)
Splicing	97	85.7 (16.0)	87.3 (16.4)	87 (16.2)
Nonsense	129	88.0 (14.2)	91.7 (14.4)	87.8 (14.2)
MI	38	85.7 (18.8)	86.8 (17.2)	87.4 (18.4)
CMD	25	72.8 (9.9)	77.4 (10.5)	73.2 (10.9)

*CMD* chromosomal microdeletion, *FSIQ* full-scale intelligence quotient, *MI* miscellaneous intragenic, *PIQ* performance intelligence quotient, *VIQ* verbal intelligence quotient.

**Fig. 2:**
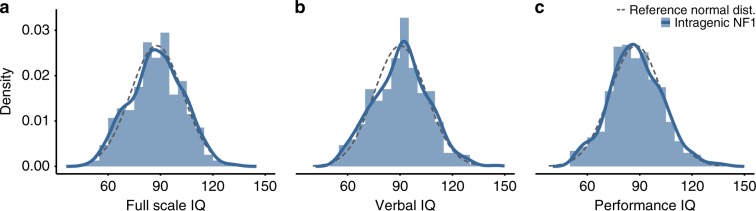
Distribution of IQ scores in neurofibromatosis type 1 (NF1) individuals with intragenic genotypes. Density plots and histograms for full-scale IQ (FSIQ) (**a**), verbal IQ (VIQ) (**b**), and performance IQ (PIQ) (**c**). Solid blue lines and light blue bars show the distribution in NF1 individuals with intragenic genotypes (no chromosomal microdeletions [CMD]; *n* = 470). Gray dashed lines are reference normal distributions with the same mean as in the NF1 data and an SD of 15.

To assess whether specific intragenic *NF1* variants increase the variability of cognitive ability relative to the general population, we characterized the variability for all children in the combined cohort with an intragenic genotype, thus excluding those with a CMD genotype. The mean FSIQ scores were lower than expected from the general population, whereas the SD was similar: mean FSIQ was 87.8 (SD 15.1), mean VIQ was 90.6 (SD 15.6), and PIQ was 87.9 (SD 15.1). Visual inspection (Fig. 2) and distribution statistics indicated a normal distribution for all three IQ scores: FSIQ (*p*_Shapiro–Wilk_ = 0.17, *z*_skewness_ = −0.11, *z*_kurtosis_ = −1.71), VIQ (*p*_Shapiro–Wilk_ = 0.11, *z*_skewness_ = 0.55, *z*_kurtosis_ = −0.67), and PIQ (*p*_Shapiro–Wilk_ = 0.06, *z*_skewness_ = 1.77, *z*_kurtosis_ = 0.50). When the CMD data were added back into the study sample (median FSIQ = 87.0 [IQR = 76.0–97.0]; median VIQ = 91.0 [79.5–99.5]; median PIQ = 86.0 [77.0–98.0]), there was a significant deviation from the normal distribution, in line with CMD children showing significantly lower IQ scores: FSIQ (*p*_Shapiro–Wilk_ = 0.07, *z*_skewness_ = 0.28, *z*_kurtosis_ = −1.98), VIQ (*p*_Shapiro–Wilk_ = 0.08, *z*_skewness_ = 0.91, *z*_kurtosis_ = −0.79), and PIQ (*p*_Shapiro–Wilk_ = 0.04, *z*_skewness_ = 1.97, *z*_kurtosis_ = 0.24). Additionally, we compared variances between the different intragenic genotype groups (missense, frameshift, splicing, nonsense, and miscellaneous intragenic, as defined in “Materials and Methods.”). These groups did not differ for FSIQ (F[4463] = 1.95, *p* = 0.10) and PIQ (F[4465] = 1.15, *p* = 0.33), but did for VIQ (F[4465] = 2.75, *p* = 0.03). However, FDR-corrected post hoc tests of variances for VIQ did not yield any significant comparisons between genotype group pairs (Table S2).

**Fig. 3:**
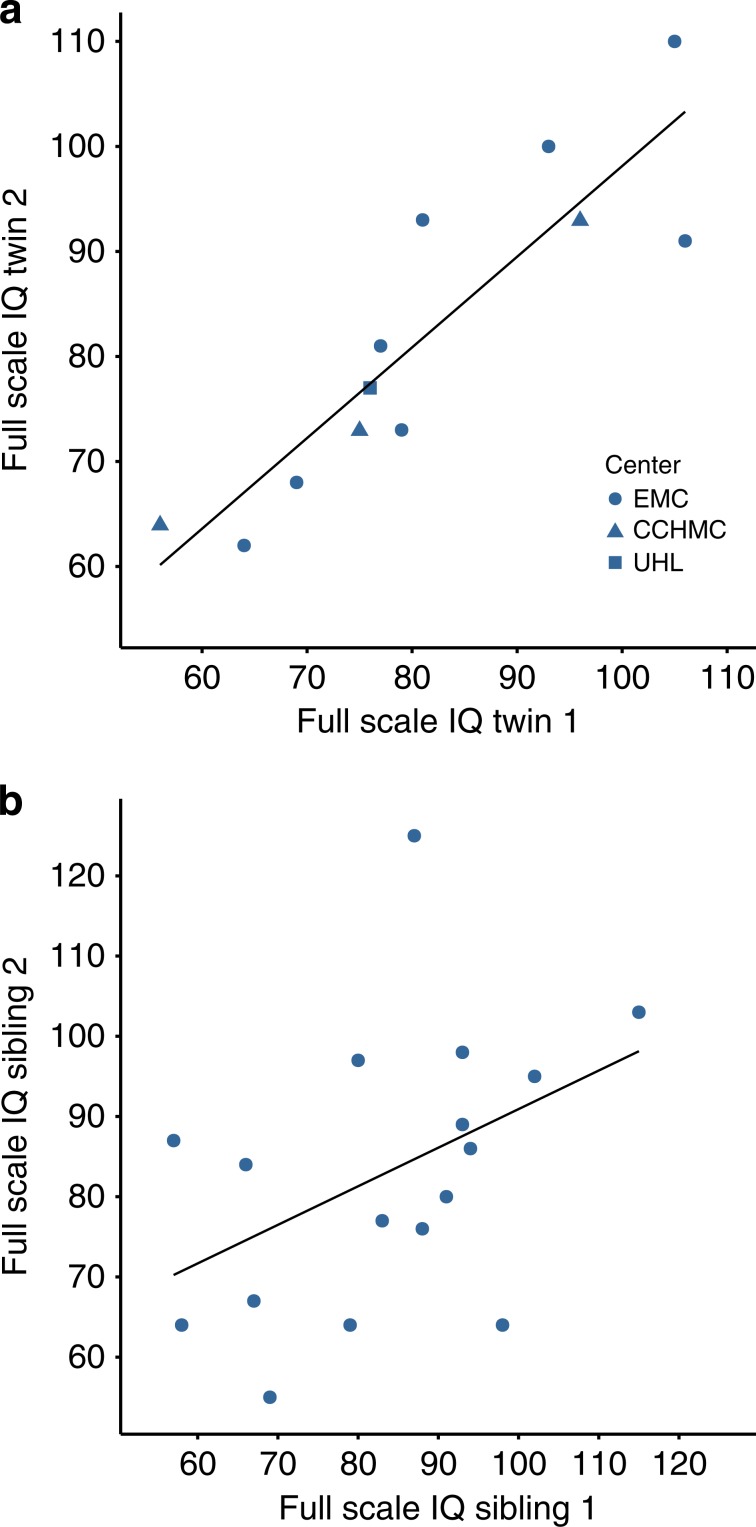
Correlation of full-scale IQ (FSIQ) within neurofibromatosis type 1 (NF1) monozygotic twin pairs. Scatterplots showing the correlation of FSIQ within monozygotic (MZ) NF1 twins (*n* = 12 pairs) (**a**) and sibling sets (*n* = 17 sets) (**b**). For the sibling sets that have 3 siblings (2/17 sets), the two individuals with the highest and lowest FSIQ score are plotted. The solid lines represent the best linear fit. The different shapes indicate whether the data were collected at the Erasmus Medical Center (EMC), Cincinnati Children’s Hospital Medical Center (CCHMC), or University Hospital Leuven (UHL).

A meta-analysis of MZ twin pair studies in the general population shows that the correlation of FSIQ between twins is very high (*r*_ICC_ = 0.85) and is mostly determined by their genetic makeup.^33^ If LOH or T2Hs played a significant role in increasing the variability, this correlation would be expected to be lower in NF1 MZ twin pairs relative to MZ twins in the general population, as these factors are considered to be noninherited, nonshared factors. We, therefore, studied the correlation of FSIQ within 12 NF1 MZ twin pairs (median age = 18.5 years [range = 4–27]; 10 female pairs; all reared together; all had an intragenic *NF1* pathogenic variant). The median FSIQ in this sample was 78 (range = 56–110; lower quartile = 72; upper quartile = 93). We found a high correlation of FSIQ within pairs (*r*_ICC_ = 0.90 [bootstrapped 95% CI = 0.71–0.96]) (Fig. 3a), which is similar to the general adult population (*r*_ICC_ = 0.85).^33^ Hence, these results indicate that the influence of noninherited, nonshared factors such as LOH is small or nonexistent.

In addition to the twin case series, we performed an exploratory analysis on 17 sibling sets who we were able to identify in our retrospective cohort collected from the EMC. We selected only those sibling sets who had an intragenic *NF1* pathogenic variant (15 pairs and 2 sets of three siblings; median age = 8.5 years [range = 4.0–14.0]; 50.0% female; none were dizygotic twins). The median FSIQ in this sample was 85 (range = 55–125; lower quartile = 69; upper quartile = 94). Within sibling sets, we found a correlation of *r*_ICC_ = 0.52 (bootstrapped 95% CI = 0.19–0.75) (Fig. 3b), which is close to that of the general population (*r*_ICC_ = 0.46).^33^ These results are in line with the normal distribution and normal degree of variation of IQ scores found in the intragenic NF1 cohort.

**Fig. 4:**
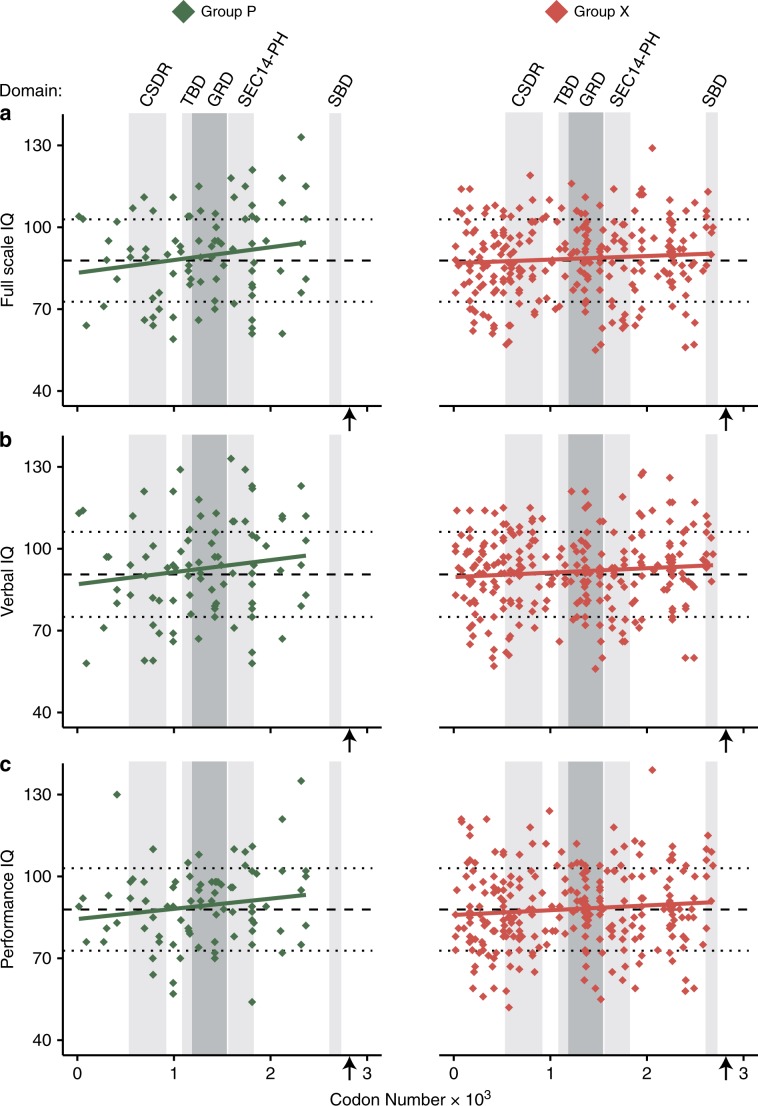
Relationship between *NF1* variant location and IQ. The scatterplot shows the relationship between the location of variants along the gene and full-scale IQ (FSIQ) (**a**), verbal IQ (VIQ) (**b**), and performance IQ (PIQ) (**c**) for group P (*n* = 84; missense variants and small in-frame deletions or insertions) and group X (*n* = 262; frameshift and nonsense variants) respectively. Long dashed line indicates the IQ mean of the subset of NF1 individuals shown; short dashed lines indicate its +1 SD and −1 SD. Gray vertical bars represent where known domains are located along the gene, with the darker gray bar indicating the GRD. The arrows indicate the C-terminal amino acid (corresponding to codon 2818). *CSRD* cysteine and serine rich domain, *GRD* GAP-related domain, *SEC14-PH* Sec14 homology-like and Pleckstrin homology-like domain, *SBD* syndecan binding domain, *TBD* tubulin-binding domain.

Lastly, as the severity of certain NF1 characteristics is related to the specific location of a variant along the gene^26–29^ and one study showed that the severity of autism symptoms showed a linear relation with variant location along the gene,^38^ we performed exploratory analyses on the location-specific effect of intragenic genotypes. We assumed that missense variants and small in-frame deletions or insertions allow expression of neurofibromin, the *NF1* gene product (group P; *n* = 84), and could possibly be more susceptible to the location of the variant location as opposed to frameshift and nonsense variants that are likely to result in absence of neurofibromin due to nonsense-mediated decay (group X; *n* = 262). Because the effects of splice variants on protein production are often difficult to predict, we excluded these variants from our analysis. Visual inspection of the relation between FSIQ and variant location along the gene did not highlight specific regions, though it did suggest a subtle positive relation between IQ score and the proximity of the variant toward the 3'-end of the gene (Fig. 4). However, a model with fixed effects for variant location, neurofibromin expression group (P or X), and their interaction explained very little variation in either IQ score, as it did not provide a better fit than an intercept-only model for FSIQ (*LR*[3] = 3.31; *p* = 0.35; *f*^2^ = 0.009), VIQ (*LR*[3] = 4.84; *p* = 0.18; *f*^2^ = 0.013), and PIQ (*LR*[3] = 3.60; *p* = 0.31; *f*^2^ = 0.010). Moreover, there was no significant effect of variant location or the interaction between variant location and neurofibromin expression group on either of the IQ scores (Table S3). A previous report showed a relation between variant location and the severity of autism symptomatology when excluding variants residing in the GAP-related domain (GRD).^38^ Therefore, we added GRD location as a variable to our model. However, this extended model did not show a significant model fit over an intercept-only model for either FSIQ (*LR*[7] = 5.88; *p* = 0.55; *f*^2^ = 0.016), VIQ (*LR*[7] = 6.49; *p* = 0.48; *f*^2^ = 0.017), or PIQ (*LR*[7] = 5.83; *p* = 0.56; *f*^2^ = 0.016), nor a significant effect of GRD location on either FSIQ (*LR*[4] = 2.57; *p* = 0.63; *f*^2^ = 0.007), VIQ (*LR*[4] = 1.65; *p* = 0.80; *f*^2^ = 0.004), or PIQ (*LR*[4] = 2.23; *p* = 0.69; *f*^2^ = 0.006). Additionally, there was no significant effect of variant location or the interaction between variant location and neurofibromin expression group within this extended model (Table S3). Together, these nonsignificant effects in the extended model indicate that the relation between cognitive ability and variant location is not dependent on the variant residing in the GRD.

## DISCUSSION

Here we studied the variability of the cognitive phenotype in NF1 and explored the extent of several genotype–phenotype correlations. This is the largest study characterizing global cognitive function in NF1 to date, and the first study relating a continuous cognitive outcome to different genotypes observed in NF1 individuals. It is also the first study quantifying the correlation of a continuous measure of cognitive function within NF1 MZ twin pairs. While the mean IQ scores were overall lower than scores in the general population, and comparable and consistent with IQ scores found in previous NF1 studies, we found that the variability of global cognitive functioning in NF1 individuals with an intragenic genotype resembles that of the general population. Additionally, we found that the intraclass correlation of IQ scores within NF1 MZ twin pairs and sibling sets is similar to correlations found in the general population. Further, we confirmed that individuals with CMDs have a lower cognitive ability than individuals with intragenic *NF1* pathogenic variants. We did not identify any genotype–phenotype correlations within the intragenic variant group, neither based on variant type, nor on the location of the variant along the gene.

The normal distribution of IQ scores in children with NF1 in combination with the high correlation of FSIQ within NF1 MZ twins and moderate correlation within NF1 sibling sets indicates that the variability of the cognitive phenotype in NF1 individuals is not higher than the unaffected population. Specifically, the normal distribution and the moderate correlation within sibling sets suggest that genetic modifiers have a similar influence on cognition in NF1 individuals as in the general population.^15,16^ Furthermore, the high correlation within NF1 MZ twin pairs suggests that NF1-specific mechanisms that can be considered to be nonshared environmental factors do not play a significant role in the cognitive function of NF1 individuals. These NF1-specific mechanisms include second-hit variants in the unaffected NF1 allele, leading to LOH, that are an important driver for the variability of several somatic NF1 features,^14^ and may include T2Hs as seen on brain MRIs. The latter would, however, be in conflict with a slight majority of studies that concluded that T2Hs associated with NF1 have a negative impact cognitive ability.^39–42^ However, if the presence of T2Hs shows a high concordance within 11 NF1 MZ twin pairs as previously suggested,^12^ it is debatable whether presence of T2Hs should be considered a nongenetic factor in the first place. Lastly, while allelic imbalance was observed in neurons derived from induced pluripotent stem cells of NF1 individuals and correlated with dopamine signaling,^19^ our results collectively indicate that allelic imbalance is unlikely to add significantly to the variability of the cognitive NF1 phenotype.

We recognize that the sample size of the twin case series was small. Nonetheless, several factors support the reliability of the observed *r*_ICC_ for FSIQ. First, we did not observe any outliers in our data set, identified as being lower than the lower quartile minus 1.5 times the IQR or higher than the upper quartile plus 1.5 times the IQR, that could compromise the accuracy of our found *r*_ICC_s. The degree of accuracy is also reflected in the relatively narrow bootstrapped 95% CI. Second, our participants were rather young, and it is known that the heritability of IQ in children is smaller than in adults,^43^ therefore our estimated *r*_ICC_ is rather conservative. Additionally, we recognize that the sample size of the sibling set case series is small. Although the *r*_ICC_ found here (*r*_ICC_ = 0.52) was reasonably close to the *r*_ICC_ of sibling sets in the general population (*r*_ICC_ = 0.46)^33^ and we did not observe any outliers in this subset either, the 95% CI was (as expected) much broader than in the twin case series. The sibling *r*_ICC_ should, therefore, be interpreted with more caution.

The only genotype–phenotype correlation we found confirmed that, as a group, children with NF1 with CMD have lower cognitive abilities.^23,24^ In line with this finding, we could show that including individuals with the CMD genotype in our distribution analysis resulted in a deviation from the normal distribution. This decreased cognitive ability in CMD individuals is believed to arise from the codeletion of genes adjacent to *NF1*, such as *OMG* (OMIM 164345) and *RNF135* (OMIM 611358).^44^ Additionally, we could not find evidence for other genotype–phenotype correlations among children with pathogenic intragenic variants: neither for intragenic genotype groups nor for gene location-specific genotypes. Lastly, our results did not show a linear relation between cognitive ability and the proximity of the variant to the 3’-end of the gene, as is suggested for autistic traits in NF1 by Morris et al.^38^ Together with the normal distribution and variation in our intragenic NF1 cohort and the lack of significant differences in the variability of IQ scores between intragenic genotype groups, these findings indicate that if location-specific genotype–phenotype correlations are to be identified in the future, they will probably account for a very small proportion of NF1 individuals.

Nevertheless, intragenic genotype–phenotype correlations concerning a single or a few neighboring amino acids (i.e., p.844–848, p.Met992del, p.Met1149, p.Arg1276, p.Lys1423, and p.Arg1809) have been identified in the context of somatic NF1 features.^26–29,45^ While theoretically one would only need a sample size of *n* = 102 for a comparison of these six specific genotypes alongside a general group with other missense pathogenic variants (assuming a medium effect size of *f*^2^ = 0.15, one categorical fixed effect with four categories, a significance criterion of α = 0.05, and a power of 1–β = 0.80), one would also need a reasonable number of individuals per group. As our data included only *n* = 2 p.844–848, *n* = 5 p.Met992del, *n* = 1 p.Met1149, *n* = 3 p.Arg1276, *n* = 4 p.Lys1423, and *n* = 9 p.Arg1809 individuals, we regarded our data as unsuitable for such an analysis. Furthermore, our study was not powered to screen for other genotype–phenotype correlations or to quantify the explained variation by intragenic genotype–phenotype correlations with locational specificity at the single amino acid level. Considering that LOVD reports 152 different amino acid locations for pathogenic missense *NF1* variants alone, one would need a database with a sample size of at least *n* = 462 (*f*^2^ = 0.15, a significance criterion of α = 0.05, and a power of 1–β = 0.80) and a reasonable number of individuals per each of those 152 location categories. This is very hard to achieve, since a recent study using the largest *NF1* gene variant database to date (8000 individuals) showed that a combination of the six most common recurrent missense or in-frame mutational hotspots (i.e., p.844–848, p.Met992del, p.Met1149, p.Arg1276, p.Lys1423, and p.Arg1809) represents only 4.8% of unrelated probands.^45^

There are several clinical implications of our findings. First of all, we provide a robust confirmation that the chromosomal microdeletion genotype is associated with a lower cognitive ability compared with intragenic NF1 genotypes. Individuals with intragenic *NF1* variants who score >1.5–2 SD below the NF1 population mean should be screened for additional variants that may explain the reduced intellectual disability, similar to current guidelines for the general population. Additionally, as we observe a downward shift of IQ relative to the general population, while the variability remains similar, our findings suggest that cognitive ability is likely to be similarly affected in all NF1 individuals with an intragenic genotype, independent of whether their IQ is within the normal range or not. Hence, for clinical trials that aim to improve cognitive ability in NF1, inclusion of subjects who perform in the normal range will likely not attenuate the treatment effect. Lastly, our data suggest that there is no major role for NF1-specific genetic or nongenetic modifiers in determining the severity of the cognitive phenotype of individuals with NF1. Rather, cognitive ability in NF1 mainly varies due to normal variation in genetic background as it does for the general population.

## Supplementary information

Supplemental Data
