# Supplementary material for: Examination of the genetic factors underlying the cognitive variability associated with neurofibromatosis type 1
Source: Genet Med. 2020 Feb 4;22(5):889–97. doi: 10.1038/s41436-020-0752-2 (PMC7200599; doi:10.1038/s41436-020-0752-2)
Supplement: Supplementary file 1 — Supplemental Data [file 41436_2020_752_MOESM1_ESM.docx]

**Supplemental Data**

|  | **Missense** | **Frameshift** | **Splicing** | **Nonsense** | **MI** |
| --- | --- | --- | --- | --- | --- |
| **FSIQ** |  |  |  |  |  |
| Frameshift | 0.88 | - | - | - | - |
| Splicing | 0.27 | 0.27 | - | - | - |
| Nonsense | 0.71 | 0.79 | 0.39 | - | - |
| MI | 0.39 | 0.39 | 1.00 | 0.54 | - |
| CMD | <0.0001 | <0.0001 | 0.0005 | <0.0001 | 0.002 |
| **VIQ** |  |  |  |  |  |
| Frameshift | 0.67 | - | - | - | - |
| Splicing | 0.046 | 0.06 | - | - | - |
| Nonsense | 0.67 | 0.99 | 0.06 | - | - |
| MI | 0.08 | 0.12 | 0.94 | 0.12 | - |
| CMD | 0.0001 | 0.0001 | 0.02 | 0.0001 | 0.046 |
| **PIQ** |  |  |  |  |  |
| Frameshift | 0.91 | - | - | - | - |
| Splicing | 0.91 | 0.91 | - | - | - |
| Nonsense | 0.91 | 0.91 | 0.91 | - | - |
| MI | 0.91 | 0.91 | 0.91 | 0.91 | - |
| CMD | <0.0001 | <0.0001 | 0.0002 | <0.0001 | 0.0007 |

**Table S1: FDR adjusted *p*-values of pair-wise comparisons of IQ scores between genotype groups.**

FSIQ, Full Scale Intelligence Quotient; VIQ, Verbal Intelligence Quotient; PIQ, Performance Intelligence Quotient; MI, miscellaneous intragenic; CMD, chromosomal microdeletion.

|  | **Missense** | **Frameshift** | **Splicing** | **Nonsense** |
| --- | --- | --- | --- | --- |
| **Missense** | - | - | - | - |
| **Frameshift** | 0.07 | - | - | - |
| **Splicing** | 0.57 | 0.12 | - | - |
| **Nonsense** | 0.12 | 0.79 | 0.21 | - |
| **MI** | 0.79 | 0.12 | 0.79 | 0.21 |

**Table S2: FDR-adjusted *p*-values for pair-wise Levene’s tests of VIQ between genotype groups.**

VIQ, Verbal Intelligence Quotient; MI, miscellaneous intragenic.

|  | **Effect variant location** | | | **Effect interaction variant location and neurofibromin expression group** | | | **Coefficients mutation location‡** | |
| --- | --- | --- | --- | --- | --- | --- | --- | --- |
|  | **LR(df)** | ***p*** | ***f^2^*** | **LR(df)** | ***p*** | ***f^2^*** | **Group P (95% CI)** | **Group X (95% CI)** |
| **Original model*** | | | | | | | | |
| FSIQ | 3.00(2) | 0.22 | 0.008 | 2.81(1) | 0.09 | 0.008 | 4.62 (-0.81-10.05) | 0.06 (-0.74-0.86) |
| VIQ | 4.47(2) | 0.11 | 0.012 | 1.83(1) | 0.18 | 0.005 | 3.85 (-1.75-9.45) | 0.57 (-0.25-1.4) |
| PIQ | 3.02(2) | 0.22 | 0.008 | 2.32(1) | 0.13 | 0.006 | 4.16 (-1.23-9.55) | -0.42 (-1.22-0.37) |
| **Controlling for GRD location †** | | | | | | | | |
| FSIQ | 5.35(4) | 0.25 | 0.015 | 3.47(2) | 0.18 | 0.009 | 4.7 (-0.76-10.17) | 0.08 (-0.72-0.88) |
| VIQ | 5.74(4) | 0.22 | 0.015 | 2.32(2) | 0.31 | 0.006 | 3.91 (-1.73-9.55) | 0.58 (-0.25-1.41) |
| PIQ | 4.88(4) | 0.30 | 0.013 | 2.59(2) | 0.27 | 0.007 | 4.22 (-1.21-9.65) | -0.41 (-1.21-0.38) |

**Table S3: The effect of variant location on IQ scores.**

FSIQ, Full Scale Intelligence Quotient; VIQ, Verbal Intelligence Quotient; PIQ, Performance Intelligence Quotient; GRD, GAP-related domain.

***** Linear mixed model with IQ score as independent variable; variant location, protein production group (P or X) and its interaction as fixed effects; and intercepts per center as random effect)

**†** The original model controlled for the variant being situated within or outside of the GRD, by adding a fixed effect for GRD location and the interaction between GRD location and the fixed effects in the original model.

‡ Coefficients are change in IQ score per 1000 amino acids in the direction of the ‘3-end of the gene. For the GRD model, the coefficients indicate the slope of either group outside the GRD.
